# Evaluating the Clinical Success of Clear Aligners for Rotational Tooth Movements in Adult Patients: A Systematic Review

**DOI:** 10.3390/dj13100440

**Published:** 2025-09-24

**Authors:** Giulia Benedetti, Nicolò Sicca, Gaia Lopponi, Claudia Dettori, Alessio Verdecchia, Enrico Spinas

**Affiliations:** 1 Department of Surgical Sciences, Postgraduate School in Orthodontics, University of Cagliari, 09124 Cagliari, Italy; giulia.benedetti995@gmail.com (G.B.); n.sicca@studenti.unica.it (N.S.); gaia.lopponi@gmail.com (G.L.); 2 Department of Surgical Sciences, School of Dental Medicine, University of Cagliari, 09124 Cagliari, Italy; claudia.dettori@gmail.com; 3 Orthodontics Division, Instituto Asturiano de Odontologia, Universidad de Oviedo, 33006 Oviedo, Spain

**Keywords:** clear aligner therapy, Invisalign, rotations, orthodontic movement, attachment

## Abstract

**Objectives:** Despite the widespread adoption of clear aligner therapy (CAT), its effectiveness in managing rotations remains debated. This systematic review aims to evaluate rotational accuracy in adults and the influence of treatment variables—such as attachments, interproximal reduction (IPR), and staging. **Methods**: Following PRISMA guidelines, seven databases and two grey literature sources were searched up to July 2025. Eligible studies assessed rotational accuracy in patients treated exclusively with clear aligners, using 3D digital model superimposition. Primary outcomes included percent accuracy, lack of correction (LC), or mean absolute error (MAE). Risk of bias (RoB 2, ROBINS-I) and certainty of evidence (GRADE) were assessed. **Results**: Twelve studies (one RCT, eleven non-randomized) were included, showing wide heterogeneity in aligner systems, tooth types, outcome measures, and adjunctive strategies. Reported accuracy ranged from 36% to 85%, averaging around 65%. LC values varied from 0.7° to 4.5°, and mean MAE was about 2.3°. Incisors and molars showed higher predictability, whereas maxillary canines and premolars remained the least reliable. Attachments and IPR were widely used, but their effectiveness was inconsistent. Staging protocols were generally set at 2°/aligner and most studies adopted 7–14-day wear schedules. Nearly all investigations showed moderate-to-serious risk of bias, and certainty of evidence was rated low to moderate. **Conclusions**: CAT shows limited yet improving predictability in rotational movements, with performance strongly influenced by tooth morphology and staging. Attachments, IPR, and overcorrections may contribute but lack consistent validation. Given the low certainty and high risk of bias of current evidence, these findings should be interpreted cautiously. Well-designed RCTs with standardized protocols are required to develop reliable clinical guidelines.

## 1. Introduction

Clear aligner therapy (CAT) has rapidly evolved over the past two decades, emerging as one of the most significant innovations in contemporary orthodontics [1,2,3]. First introduced in the late 1990s with the advent of digital treatment planning and CAD/CAM manufacturing, aligners have progressed from an esthetic solution for mild malocclusions to a mainstream option for managing increasingly complex cases [4,5,6,7,8,9].

Their widespread adoption is largely attributed to advantages such as improved esthetics and comfort, reduced pain, minimal interference with speech, and easier maintenance of oral hygiene due to their removability [10,11,12,13,14]. For clinicians, clear aligners also offer practical benefits, including reduced chair time, fewer emergencies, and extended intervals between visits compared to fixed appliances [15,16,17,18].

Nonetheless, CAT is not without limitations. It requires high patient compliance and is associated with increased production costs [18,19]. Moreover, several biomechanical limitations remain unresolved, with aligner performance still lagging behind that of braces [8,9,20]. Although manufacturers claim the ability to achieve complex movements, clear aligners struggle to manage complex movements such as extrusion, torque, root translation, and rotation [2,21,22]. The latter are especially challenging in teeth with rounded cross-sections (e.g., canines and premolars), often resulting in poor correspondence between simulated and achieved movement. Such discrepancies not only prolong treatment time but may also lead to patient dissatisfaction and increased risk of relapse [23].

These concerns were underscored in a previous systematic review with meta-analysis by Koletsi et al. [24] who specifically addressed the effectiveness of clear aligners in achieving rotations. The authors reported suboptimal accuracy and found that neither optimized attachments nor interproximal reduction (IPR) consistently enhanced performance [24]. More recent reviews further confirmed these findings, emphasizing that staging and the magnitude of planned movement may significantly influence rotational predictability [6,22,25,26]. However, the clinical applicability of these observations is limited by the scarcity of high-quality controlled clinical trials comparing CAT with traditional orthodontic techniques [27]. As a result, clinicians must often rely on limited evidence, expert opinion, and anecdotal experience in their decision-making process [28].

At the same time, research on CAT has seen exponential growth, as evidenced by bibliometric analyses showing increased publication and citation rates in recent years [4,29,30]. In light of this evolving context, we deemed it timely to conduct an updated systematic review, with the primary aim of assessing the predictability of clear aligners in achieving rotational tooth movements in clinical settings. Secondary objectives include evaluating the potential influence of adjunctive strategies—such as attachments, IPR, and staging protocols—on treatment outcomes. The findings are expected to provide evidence-based guidance for optimizing treatment planning.

## 2. Materials and Methods

### 2.1. Protocol and Registration

This systematic review was carried out in accordance with the Preferred Reporting Items for Systematic Reviews and Meta-analysis (PRISMA) guidelines [31] and submitted to PROSPERO on 14 April 2025 with registration number CRD420251032934. The full review protocol was written in advance and may be accessed through this link: https://www.crd.york.ac.uk/PROSPEROFILES/625bd6a263db797c641756c77d058e51.pdf (accessed on 14 April 2025).

The research team developed a PICO question to find relevant studies in the literature:

- Population: orthodontic patients undergoing CAT;
- Intervention: predicted rotational tooth movement on the virtual treatment plan;
- Comparison: achieved rotational tooth movement;
- Outcome: accuracy of the rotation.

### 2.2. Search Strategy

A comprehensive literature search was performed between 15 April and 24 July 2025 by two researchers independently (G.B., N.S.) to identify articles relevant to the study question. The following electronic databases were investigated: PubMed, Scopus, Embase, Web of Science, Cochrane Library, LILACs, and Ovid. In addition, portals as ClinicalTrials.gov and ProQuest.com were screened to retrieve relevant grey literature.

The search strategy was developed according to the PICO framework, combining MeSH terms and free-text keywords. It was first developed for PubMed and subsequently tailored to the other databases. Detailed queries are reported in Table 1.

Finally, a manual search of the reference lists from all included studies and relevant reviews was performed to identify further eligible articles.

### 2.3. Eligibility Criteria

The eligibility criteria were defined according to the study goals and the PICO question.

Papers addressing rotational tooth movements with any type of aligner brand were selected. Both studies focused exclusively on rotations and those analyzing multiple tooth movements were considered, provided they assessed the discrepancy between predicted and achieved outcomes through superimposition of initial, planned, and final models. Rotations had to be digitally planned and quantitatively defined before treatment initiation using orthodontic software. Only results measured after the first set of aligners were deemed valid, whereas data obtained following refinements were excluded. This decision was based on the rationale that refinements are typically prescribed to compensate for discrepancies between planned and achieved movements. Including them would therefore overestimate the predictability of the initial digital treatment plan, which represented the primary focus of the review.

Studies using appliances other than CAT (e.g., fixed buccal or lingual appliances) or hybrid approaches with additional mechanics—such as power chains, intermaxillary elastics, or temporary anchorage devices (TADs)—were excluded. Adjunctive procedures like piezocision or electropulse stimulation were also deemed ineligible, while attachments and IPR were allowed as auxiliary tools.

Included articles had to involve adult patients (≥18 years) with full permanent dentition. Those dealing with adolescent patients, mixed dentition, and individuals affected by periodontitis or craniofacial syndromes were excluded. Pre-surgical and extraction-based treatments were also not considered.

Accepted study designs comprised both randomized controlled trials (RCTs) and non-randomized investigations, such as cohort, case–control, cross-sectional, and longitudinal observational studies, whether prospective or retrospective. Conversely, the following types of publications were excluded: reviews of any kind (systematic, scoping, narrative), meta-analyses, animal or in vitro studies, finite element analyses, case reports, case series, expert commentaries, letters to the editor, and abstracts only. No restrictions were applied regarding language or publication date.

**Figure 1:**
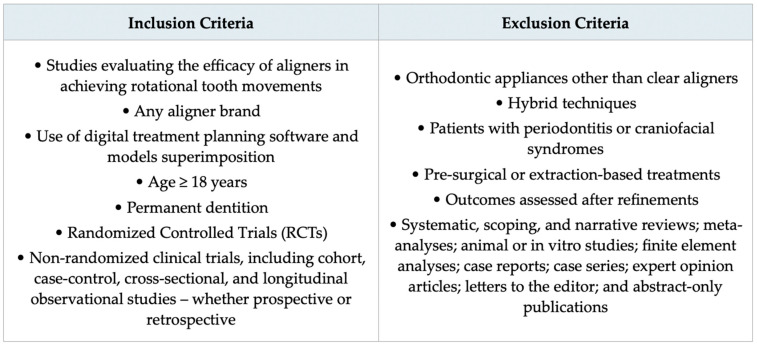
Figure showing the eligibility criteria used for this review.

The inclusion and exclusion criteria for admittance in the systematic review are summarized in Figure 1.

### 2.4. Selection of Sources of Evidence

Three reviewers (G.B., N.S., G.L.) independently screened the search results.

Duplicate records were removed using Zotero (version 6.0.36) and manually verified for accuracy. A document listing all eligibility criteria was prepared to streamline the selection process. Titles and abstracts were first assessed, and full texts of potentially relevant articles were then retrieved for detailed evaluation. The final set of included studies was established after this review.

Disagreements were resolved through discussion to reach consensus or alternatively by consulting a fourth expert researcher (E.S.). Cohen’s K coefficient [32] for agreement between the reviewers was 0.89.

### 2.5. Risk of Bias Assessment

Two researchers (G.B., A.V.) separately assessed the risk of bias of the included studies in accordance with Cochrane guidelines. The Risk of Bias (RoB 2.0) tool was applied to randomized controlled trials [33], while the Risk of Bias in Non-randomized Studies of Interventions (ROBINS-I) tool was used for non-randomized clinical investigations [34]. Discrepancies were resolved through discussion or, if needed, by consulting a third author (E.S.).

For randomized studies, five domains were evaluated and rated as low-risk, some concerns, or high risk of bias. For non-randomized trials, seven domains were assessed within the ROBINS-I framework and classified as low-, moderate-, serious-, or critical-risk, or marked as having insufficient information.

The overall certainty of evidence was further appraised using the GRADE (Grading of Recommendations Assessment, Development and Evaluation) methodology [35,36,37]. This system grades evidence as high, moderate, low, or very low, based on risk of bias, inconsistency, indirectness, imprecision, and potential publication bias. Non-randomized studies start at a low certainty level by default but may be upgraded in the presence of strong effects, evidence of a dose–response relationship, or plausible residual confounding likely to reduce the observed effect. Conversely, serious limitations in any domain—except publication bias, which is either suspected or not detected—may lead to downgrading.

### 2.6. Data Charting Process

Four authors (G.B., N.S., G.L., C.D.) collaboratively developed an Excel spreadsheet to systematically extract data from the included studies. The following variables were collected:

- First author and year of publication, country, study design, sample size, average age, and participant gender;
- Aligner system, teeth assessed, superimposition software, mean planned and achieved rotation, and accuracy of movement;
- Use and type of attachments, presence of IPR, mean number of aligners, staging protocol, wear schedule (days), and mean treatment duration.

The primary outcome was the accuracy of rotational movement, expressed as percent accuracy, lack of correction (LC), or mean absolute error (MAE), depending on the methodology of each study.

Data charting was performed iteratively, with continuous refinement to ensure accuracy, consistency, and completeness.

### 2.7. Dealing with Missing Data

Corresponding authors were contacted via e-mail to retrieve missing data when necessary. In the absence of a response or failure to supply the requested information, only the available reported data were included in the analysis.

### 2.8. Synthesis of Results

A narrative summary of the data from the included articles is presented. Statistical analysis was planned exclusively in the presence of considerable similarity across studies.

## 3. Results

### 3.1. Selection of Sources of Evidence

The computer-assisted search yielded 2738 articles across seven databases: PubMed (n = 674), Scopus (n = 582), Embase (n = 415), Web of Science (n = 594), Cochrane Library (n = 65), LILACs (n = 33) and Ovid (n = 240). An additional 135 records were retrieved from grey literature sources, including ClinicalTrials.gov (n = 41) and ProQuest.com (n = 94). No further studies were identified through manual searching.

After removing duplicates (n = 1427), 1311 studies remained for screening. Based on title and abstract evaluation, 1234 were excluded as either not pertinent (n = 1098) or unsuitable due to article type (n = 136). One full-text article could not be retrieved. Among the 76 studies evaluated in full, 64 were excluded for not meeting the inclusion criteria: 27 did not assess rotational performance, and 9 did not compare initial, planned, and achieved models using superimposition. Eight studies involved patients younger than 18 years, while six included either extraction or presurgical treatment protocols. Additionally, nine articles used adjunctive tools, three assessed outcomes post-refinement, and two were pilot studies.

**Figure 2:**
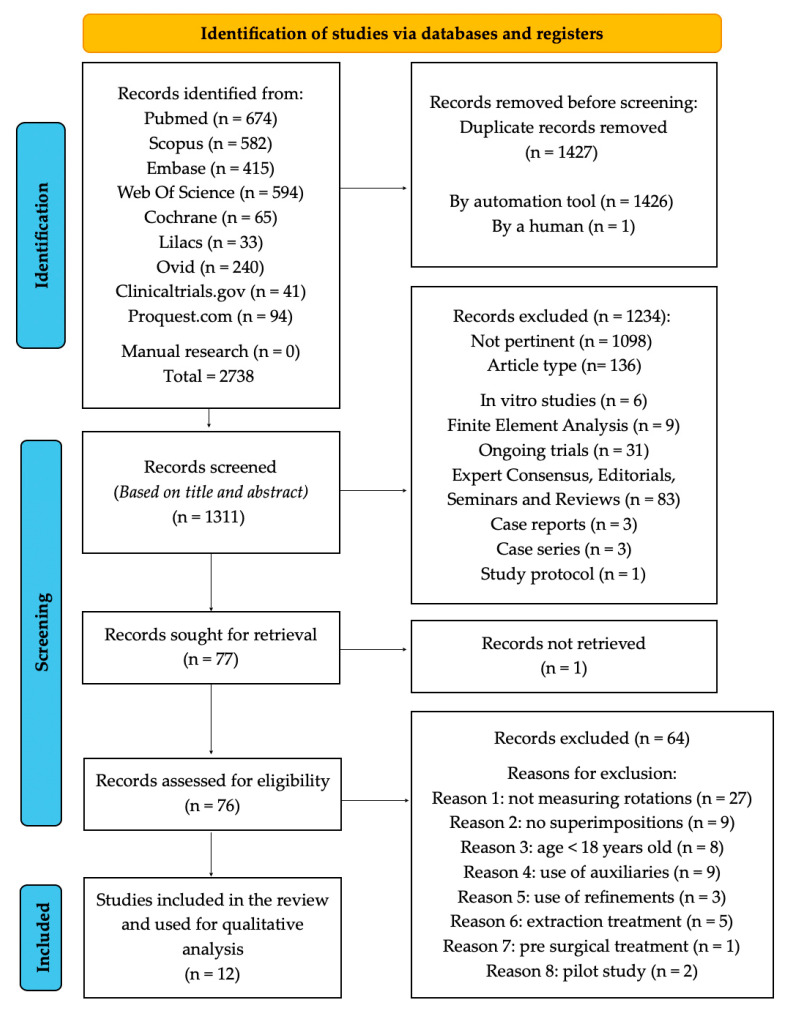
Flowchart of the study selection process.

Ultimately, 12 studies [38,39,40,41,42,43,44,45,46,47,48,49] met the eligibility criteria and were included in the systematic review. The details of the search and selection process are presented in the flowchart in Figure 2.

### 3.2. Characteristics of Sources of Evidence

Table 2 summarizes the main characteristics of the included studies, offering a comprehensive overview.

The 12 articles were conducted in six countries: Italy (n = 5) [39,40,41,43,46], USA (n = 3) [38,42,49], Australia (n = 1) [44], Brazil (n = 1) [45], Thailand (n = 1) [47], and India (n = 1) [48]. Only 1 randomized clinical trial [38] was identified, while the remaining 11 were non-randomized investigations, comprising 5 prospective [39,40,41,42,47] and 6 retrospective [43,44,45,46,48,49] studies.

**Table 2:** Main characteristics of the twelve studies included in the review.

First Author, Year (Reference)	Country	Study Design	Sample Size (n° of Patients)	Average Age (Years)	Gender of Participants
Al-Nadawi 2021 [38]	USA	RCT	89	35.4	30 M, 45 F
Castroflorio 2023 [39]	Italy	Prospective	79	30.8 ± 12	23 M, 56 F
D’Antò 2024 [40]	Italy	Prospective	45	29.2 ± 6.6	21 M, 24 F
Ghislanzoni 2024 [41]	Italy	Prospective	21	20.1 ± 1.9	9 M, 12 F
Kravitz 2008 [42]	USA	Prospective	31	29.4	13 M, 18 F
Lombardo 2017 [43]	Italy	Retrospective	16	28.7	6 M, 10 F
Maree 2022 [44]	Australia	Retrospective	30	N/R	N/R
Medeiros 2024 [45]	Brazil	Retrospective	56	33	17 M, 39 F
Mario 2024 [46]	Italy	Retrospective	120	35.2 ± 7.4	64 M, 56 F
Sachdev 2021 [47]	Thailand	Prospective	30	31.8	10 M, 20 F
Sorour 2022 [48]	India	Retrospective	62	33	19 M, 43 F
Taebi-Harandy 2023 [49]	USA	Retrospective	32	34.52 ± 11.3	3 M, 29 F

F: females; M: males; N/R: not reported.

Sample sizes ranged from 16 [43] to 120 patients [46], with mean ages varying between 20 [41] and 35 [38,46,49] years. In nearly all articles, female participants outnumbered males, except in the trial by Palone et al. [46].

### 3.3. Results of Individual Sources of Evidence

Table 3 and Table 4 synthesize the evidence related to the primary and secondary outcomes of this review. The data were divided into two tables to enhance clarity and facilitate consultation.

**Primary outcome**

#### 3.3.1. Aligner System Used

Among the 12 included studies, the most frequently used aligner system was Invisalign (Align Technology, Santa Clara, CA, USA), reported in 7 investigations [38,39,41,42,44,45,48]. Two were comparative: Medeiros et al., contrasted first-generation Invisalign (EX30) with the second-generation version (SmartTrack) [45], while Sorour et al., evaluated Invisalign against Flash aligners (Flash Orthodontics, Mumbai, India) [48].

Other aligner brands included F22 (Sweden & Martina, Due Carrare, Italy) [43,46], Ordoline Aligners (UAB Ordoline, Vilnius, Lithuania) [40], and 3M Clarity (3M, Monrovia, CA, USA) [49]. Additionally, the study by Sachdev assessed the effectiveness of an in-house, 3D-printed aligner system [47].

#### 3.3.2. Teeth Assessed

The types of teeth evaluated varied across the included studies.

Ten out of twelve investigations assessed teeth from both arches, whereas Maree et al., focused exclusively on the maxillary arch [44]. In Medeiros’ study, it was unclear whether measurements involved one or both arches, as neither Section 3 Materials and Methods nor Section 4 Results provided this information [45].

Most articles examined the full dentition, from central incisors to molars. Specifically, Ghislanzoni and Palone limited their analysis to first molars [41,46], while Al-Nadawi, Castroflorio, D’Antò, and Lombardo included second molars as well [38,39,40,43]. Medeiros did not specify whether both first and second molars were considered or only one of the two [45].

Conversely, some studies analyzed only a subset of teeth, either up to second premolars [49] or limited to canines [47,48]. Finally, two articles targeted individual teeth: Maree assessed maxillary central incisors, and Kravitz focused on canine performance in both arches [42,44].

#### 3.3.3. Superimposition Software

In accordance with the inclusion criteria of this review, only trials that evaluated aligner rotational performance through superimposition of pre- and post-treatment digital models were considered.

A variety of 3D metrology software solutions were employed across the studies. Three investigations used Geomagic Control (3D Systems, Rock Hill, SC, USA) [40,44,45], while Castroflorio adopted an earlier version, Geomagic Qualify [39]. Other groups relied on eModel Compare (GeoDigm Corporation, Falcon Heights, MN, USA) [38,48] or VAM software (Vectra, Canfield Scientific, Fairfield, NJ, USA) [41,43].

Additional programs used in individual studies included ToothMeasure (Align Technology Inc., Tempe, AZ, USA) [42], OnyxCeph^3^™ (Image Instruments GmbH, Chemnitz, Germany) [46], OrthoAnalyzer™ (3Shape, Copenhagen, Denmark) [47], and 3D Slicer (Brigham and Women’s Hospital, Boston, MA, USA) [49].

#### 3.3.4. Mean Planned Rotation and Mean Achieved Rotation

Planned rotation was defined as the angular difference between tooth position in the digital setup and its location in the pre-treatment model. All included studies, except those by Al-Nadawi and Sorour [38,48], reported mean planned values, ranging from 2.54° [41] to 18.75° [44].

In contrast, only eight out of ten articles reported the mean amount of rotation achieved after the initial set of aligners, varying between 1.56° [41] and 13.37° [44]. Achieved movement was calculated as the discrepancy between tooth position in the post-treatment scan and the initial model. In comparative trials, these values were presented separately for each treatment group.

The absolute figures mentioned above should be in light of the spatial coordinate reference systems adopted. These were explicitly described by Castroflorio, D’Antò, Ghislanzoni, Lombardo, Maree, and Medeiros [39,40,41,43,44,45], but omitted in the remaining investigations.

#### 3.3.5. Percent Accuracy

Half of the studies in this review described rotational performance in terms of the percentage accuracy of achieved versus planned movement [40,42,43,44,46,47], reporting values that ranged from 35.8% [42] to 76.8% [40]. The calculation methods, however, varied across authors.

D’Antò and Palone computed accuracy using the formula (achieved movement/predicted movement) × 100 [40,46]. Similarly, Lombardo expressed the rotational accuracy index as the ratio of achieved to prescribed movement, where values closer to 1 indicate greater precision [43]. Sachdev, on the other hand, applied a different formulation: 100 − (((predicted movement − achieved movement)/predicted movement) × 100) [47]. Both formulas are mathematically equivalent—each reflecting accuracy as a function of achieved over intended rotation. Kravitz and Maree did not specify the method used to calculate percentage accuracy [42,44].

#### 3.3.6. Lack of Correction (LC)

A second approach for evaluating rotational performance measures the discrepancy between planned and achieved movement, calculated as prescribed rotation − achieved rotation. This parameter, known as Lack of Correction (LC), reflects the extent of unexpressed movement, with lower values indicating greater accuracy.

More than half of the included studies applied this method [38,39,43,45,46,47,48,49], with reported values ranging from 0.74° [49] to 4.48° [45].

#### 3.3.7. Mean Absolute Error (MAE)

An additional method used to assess rotational accuracy, adopted in only 2 out of the 12 studies [40,41], is the Mean Absolute Error (MAE). This metric is defined as the absolute difference between the achieved and planned rotational movements, calculated as |achieved rotational movement − planned rotational movement|. Both authors reported an MAE value of 2.3°.

#### 3.3.8. Rotational Accuracy by Tooth Type

*Incisors:* Reported accuracy for central maxillary incisors ranged from 35.1% with in-house 3D-printed aligners [47] to 70.2% with Invisalign SmartTrack (San Jose, CA, USA) [44]. Similarly, Ordoline aligners achieved values above 70% [40], while the F22 system showed mean values of 61.5% and 61.28% [43,46]. For maxillary lateral incisors, Ghislanzoni observed a mean underperformance of 4.2° [41], with similar discrepancies noted by other authors [40,45,49]. When considering mandibular incisors, accuracy values ranged from 67% [43] to 90% [42].

*Canines:* The study by Kravitz et al., showed an average accuracy of 36% with Invisalign EX30, with only 15 of the 53 canines achieving correction greater than 50% [42]. Palone reported comparable results, with maxillary canines showing the lowest accuracy (~50%), and mandibular canines performing slightly better at 58% [46], a pattern also noted by Sachdev and Lombardo [43,47]. In terms of LC and MAE, every canine rotation leaves a portion of planned movement unexpressed—approximately 4.5° for maxillary and 2.5° for mandibular canines [39,41,45,48].

*Premolars:* D’Antò et al., reported mean rotational accuracy values of 81% in the maxilla and 72% in the mandible [40]. With the F22 system, performance ranged from 54% to 63% for maxillary premolars and from 54% to 83% for mandibular ones [43,46]. Castroflorio documented a Lack of Correction (LC) of 2.2° for maxillary and 3.8° for mandibular premolars [39], while Medeiros et al., reported slightly higher values, around 4.6° [45]. Ghislanzoni et al., calculated a Mean Absolute Error (MAE) of approximately 1.2° in both arches [41].

*Molars:* Accuracy for first molars generally exceeded 75%. With Ordoline aligners, mean values were 78.39% in the maxilla and 74.68% in the mandible [40], while Lombardo reported 85% and 78% using the F22 system [43]. Medeiros et al., found comparable Lack of Correction (LC) with Invisalign SmartTrack (2.27°) and EX30 (2.30°) [45]. MAE values reported by Ghislanzoni et al., for molars were 0.4° in the maxilla and 0.8° in the mandible [41]. Second molars showed slightly lower accuracy in both arches, as documented by D’Antò and Castroflorio [39,40].

**Secondary outcomes**

#### 3.3.9. Attachments

All but one study [49] reported the use of attachments as adjuncts to the treatment protocol. In terms of attachment type and configuration, most authors employed either conventional or optimized designs, tailored to patient-specific needs or tooth morphology [38,39,40,41,44]. Trials involving the F22 system implemented Grip Points with triangular geometries [43,46].

Castroflorio and Kravitz intentionally included control groups without attachments to compare rotational performance with and without these auxiliaries [39,42]. Notably, only Palone and Sachdev specified thresholds of planned rotation above which attachments would be applied: Palone used 10° for rounded teeth and 20° for mandibular and maxillary lateral incisors, whereas Sachdev set the cutoff at 5° [46,47]. Where reported, the average number of attachments ranged from 6 to 11 per arch [38,48].

#### 3.3.10. Interproximal Reduction (IPR)

Interproximal reduction (IPR) was performed in most articles, either following standardized protocols or at the clinician’s discretion. Maree et al., explicitly avoided IPR, while Kravitz et al., designed one group to evaluate its specific impact on rotational performance alongside two controls where IPR was not permitted [42,44]. When described, IPR was usually less than 1 mm per arch or confined to anterior teeth [38,41].

Three studies did not provide any information regarding the use of IPR [45,48,49].

#### 3.3.11. Rotation Rate and Mean Number of Aligners

Only a few studies quantified the rotation rate per aligner, consistently reporting 2° per stage [39,43,44,46,47].

The number of aligners required varied considerably across articles, ranging from 7 [47] to 27 [39] where specified. Four investigations did not provide information on the average number of aligners needed to complete the treatment plan [40,43,44,49].

#### 3.3.12. Days of Aligner Wear and Average Treatment Duration

The recommended aligner wear time varied between 7 and 21 days, with most authors adopting a 14-day change protocol [41,43,44,45,46,47]. Al-Nadawi stratified his sample into three groups based on different wear schedules (7, 10, and 14 days) to specifically assess the effectiveness of tooth movement under varying aligner replacement protocols [38].

Average treatment time, when reported, ranged from 5 to 11.2 months [38,39,42,45,48], with the majority of studies indicating durations around 7–9 months. This information was not disclosed in several articles [40,43,44,47,49].

### 3.4. Risk of Bias Assessment

#### 3.4.1. RoB 2

According to the RoB 2 tool [33], the only randomized controlled trial included in this review [38] was rated as having an overall risk of bias classified as “some concerns.” Specifically, low risk was identified for the randomization process, missing outcome data, outcome measurement, and selection of reported results. The domain of deviations from intended interventions was judged as “some concerns” due to limited reporting on blinding and the inability to objectively verify patient compliance with the assigned protocol.

**Figure 3:**
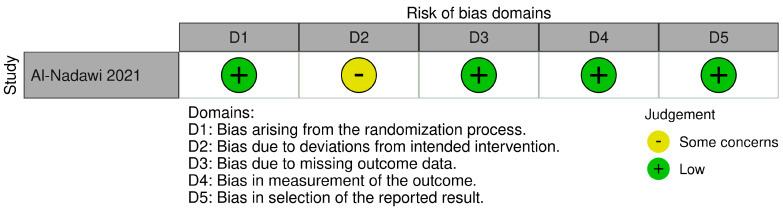
Domain-specific risk of bias assessment for the RCT by Al-Nadawi et al. [38] using the RoB 2.0 tool [33].

A visual summary of these domain-specific assessments is provided in Figure 3.

#### 3.4.2. ROBINS-I

The remaining eleven studies [39,40,41,42,43,44,45,46,47,48,49] were assessed using the ROBINS-I tool [34]. None achieved an overall low risk of bias. The most affected domains were confounding (D1), participant selection (D2), and deviations from intended interventions (D4). In particular, D4 was consistently rated as moderate or serious, reflecting the difficulty of objectively assessing patient compliance—a factor that can significantly influence the rotational performance of aligners. While some studies employed tools such as patient questionnaires to monitor aligner wear, others did not address this issue, introducing uncertainty in adherence assessment.

The domains concerning classification of interventions (D3) and outcome measurement (D6) showed variable ratings, from low to moderate risk, with one study raising serious concerns in D3. By contrast, the domains addressing missing outcome data (D5) and selection of the reported results (D7) were generally rated at low risk, indicating overall transparency and completeness in reporting.

Notably, the study by Taebi-Harandy et al. [44] exhibited the highest cumulative risk of bias, with three domains rated as serious.

**Figure 4:**
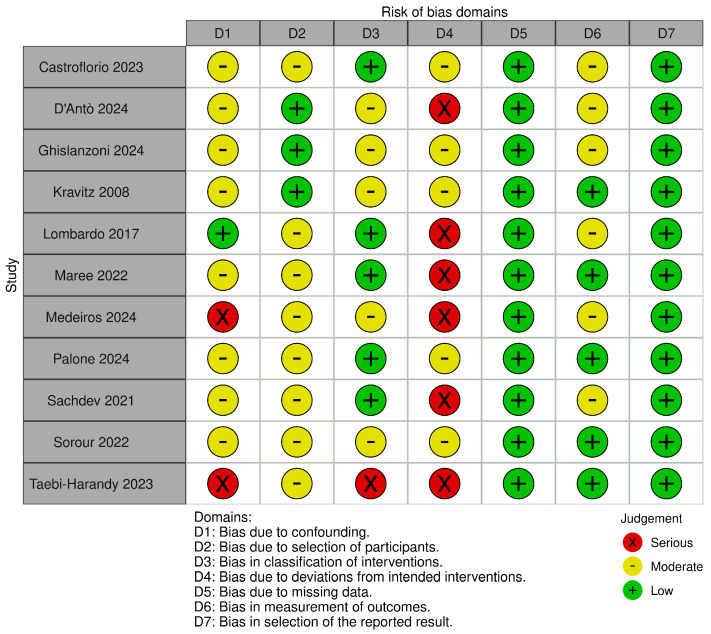
Risk of bias assessment for the eleven non-randomized studies, according to ROBINS-I, detailing judgments across seven domains [34,39,40,41,42,43,44,45,46,47,48,49].

These findings are visually summarized in the corresponding risk of bias graph (Figure 4).

#### 3.4.3. GRADE

The certainty of evidence was assessed using the GRADE approach (Table S1) [35,36,37]. The randomized controlled trial by Al-Nadawi [38] initially qualified as high-certainty was downgraded to moderate due to potential performance bias from its single-center, single-operator design and imprecision related to the small sample size (n = 75). The eleven non-randomized studies, which start at low certainty by default, remained at this level [39,40,41,42,43,44,45,46,47,48,49]. Downgrades were related to inconsistency—stemming from heterogeneity in aligner systems, tooth types, and measurement method—and to imprecision, reflected in the wide range of reported accuracy values. No upgrading criteria were met.

A Summary of Findings (SoF) table presenting the main outcomes, effect estimates, and certainty of evidence is provided in the Supplementary Materials (Table S2).

### 3.5. Synthesis of the Results

Substantial methodological heterogeneity was observed among the included studies, involving differences in aligner systems, types of teeth assessed, definitions and measurement of rotational accuracy and factors that may affect the results. The most frequently reported outcome was percent accuracy, followed by Mean Absolute Error and Lack of Correction. While the majority of studies employed attachments and IPR to facilitate rotations, only half provided details on staging protocols. The most commonly reported wear schedule was 14 days, although some authors recommended changes every 10 or even 7 days. Reported treatment durations, where available, did not exceed one year.

Given this pronounced heterogeneity in study design, outcome definitions, and reporting standards, a quantitative synthesis or meta-analysis was deemed inappropriate, as pooling the data would likely have produced misleading estimates. Therefore, the findings were synthesized narratively, allowing a more reliable appraisal of trends and limitations across the available evidence. For the same reasons, a formal assessment of reporting bias was not feasible. Nonetheless, the potential for reporting bias cannot be excluded, particularly considering the predominance of non-randomized and retrospective studies.

## 4. Discussion

### 4.1. Summary of Evidence and Comparison with Existing Literature

This systematic review aimed to evaluate the rotational accuracy of clear aligners in adult patients and to identify clinical factors influencing treatment outcomes. A total of 2.738 records were retrieved through a comprehensive search across major scientific databases and grey literature. Following a rigorous selection process, 12 studies were included for qualitative analysis. A quantitative synthesis was not performed due to the considerable heterogeneity in study designs, aligner systems, and outcome measures.

All trials consistently reported a significant discrepancy between planned and achieved rotations. Among those assessing full-arch performance, percentage accuracy ranged from 61% to 77% [40,43,46]. These findings align with earlier reports [50,51,52], suggesting that current digital treatment planning software tends to overestimate the effectiveness of rotational corrections. Clinicians should therefore anticipate a degree of unexpressed movement when interpreting virtual simulations. This discrepancy is reflected in objective parameters such as Lack of Correction (LC) and Mean Absolute Error (MAE). Reported LC values varied widely, from 0.74° to 4.48° [45,49], while the two studies calculating MAE found values of approximately 2.3° [40,41]. Rotational accuracy also appeared to differ between maxillary and mandibular arches, although findings were inconsistent. Ghislanzoni [41] suggested maxillary rotations are generally more predictable, whereas Medeiros [45] and Kravitz [42] found no significant differences, and Palone [46] reported slightly greater accuracy in the mandibular arch.

Comparable results have been reported in previous reviews, such as that by Robertson [53], who documented accuracy rates between 57% and 76%. Similarly, Koletsi’s meta-analysis found mean rotational accuracy of 48% for maxillary canines—identified as the most challenging teeth to rotate—and up to 71% for mandibular incisors, considered the most predictable [24]. Notably, neither the use of attachments nor the application of IPR significantly enhanced outcomes, and the certainty of available evidence was rated low to moderate. More recent observational studies have reported comparable or slightly higher accuracy levels [54,55,56,57].

The heterogeneity in described results likely reflects differences in tooth morphology, aligner systems, and methodological approaches [58,59]. These aspects will be explored in the following sections and compared with existing evidence.

#### 4.1.1. Tooth Type

Rotational predictability with CAT is strongly influenced by tooth morphology, particularly crown shape and root configuration [25,50]. While a few previous studies reported limited effects of tooth type on movement outcomes [52,56], the majority of evidence confirms substantial variability across dental groups: incisors and first molars generally show the highest accuracy, whereas premolars and especially canines remain the most challenging to derotate.

Incisors typically respond well to aligner-driven rotations due to their relatively flat crown and reduced root surface area, which facilitate force application and decrease resistance to movement [60,61]. However, performance differs between arches. In the maxilla, central incisors tend to be more predictable than laterals, the latter often showing reduced accuracy likely because of their intermediate position between two larger teeth, which diminishes aligner grip and compromises effective force transmission [12,57,62]. In contrast, in the mandible, lateral incisors usually perform better than centrals, possibly due to morphological differences in crown shape and root alignment, which allow more stable engagement of the aligner [40,47]. When compared with Koletsi’s meta-analysis, slightly lower values were observed in earlier literature—54.5% and 51.5% for central and lateral maxillary incisors, respectively [24]. Such differences likely reflect advancements in aligner materials, digital planning software, and clinical expertise over time [57].

Canines consistently represent the least predictable teeth to derotate, particularly in the maxillary arch [42,43,46,47]. Their rounded crowns, large root surface, and lack of undercuts hinder the formation of effective force couples, making slippage more likely. This biomechanical limitation is often described as the “watermelon seed effect,” where aligners slide over convex surfaces instead of transmitting pure rotational forces [21,22,63,64,65]. In terms of LC and MAE, canine rotations invariably leave part of the planned movement unexpressed—approximately 4.5° for maxillary and 2.5° for mandibular canines [39,41,45,48].

Premolars also show reduced accuracy, but the heterogeneity of findings prevents clear conclusions on whether maxillary or mandibular premolars are more predictable. As with canines, the combination of convex crown surfaces and relatively extensive root area increases resistance to rotational correction [2,66,67].

By contrast, molars appear more responsive to rotational forces [40,43]. A recent prospective study by Lione et al., reported a predictability rate of approximately 82% for first molar derotation [51]. The authors highlighted the value of incorporating an expansion component during digital treatment planning to create additional space and improve distal rotation, a recommendation corroborated by other studies suggesting that expansion combined with derotation enhances outcomes [68,69]. Overall, first molars achieve higher accuracy than second molars [39,40,70,71], likely due to the absence of distal anchorage and the shorter clinical crowns of second molars, which reduce aligner grip and control [72].

To sum up, these findings align with previous trials and systematic reviews [24,71,73,74,75], which consistently identified canines and premolars as the least predictable teeth to rotate, while incisors and molars showed comparatively higher accuracy. However, the present analysis also reveals a modest trend toward improved rotational performance, even in teeth historically considered the most challenging. This improvement appears to be supported by more recent studies, likely reflecting advancements in aligner materials, software, and clinical protocols [52,59,62,76].

#### 4.1.2. Attachments

Composite attachments are auxiliary features designed to enhance aligner biomechanics by increasing tooth surface contact and improving retention. They act as anchorage points for controlled force application, thereby refining the precision of tooth movement and extending the effective lever arm, particularly for complex movements such as rotations [72,77].

Two main designs are currently in use: conventional attachments, manually placed by clinicians, and optimized attachments, automatically generated by digital planning software. Optimized designs incorporate active surfaces intended to direct the magnitude and orientation of force delivery more precisely [5]. The F22 system introduced proprietary “Grip Points,” small triangular features aimed at improving rotational control [43,46].

In this review, all but one study reported the use of attachments [49]. Most employed either conventional or optimized versions tailored to tooth morphology and treatment goals [38,39,40,41,44]. Notably, Castroflorio and Kravitz included control groups without attachments to directly evaluate their contribution [39,42]. However, reported benefits were inconsistent. Some studies, such as Castroflorio [39] and Ghislanzoni [41], documented measurable advantages with optimized attachments, while others, including Medeiros [45], did not find statistically significant improvements. The limited additional effect of attachments was also reflected in comparative analyses showing no consistent difference between conventional and optimized designs [63,74,76].

Biomechanical studies have further highlighted the importance of geometry and positioning. Finite element models suggest that rectangular attachments placed on both buccal and lingual surfaces may improve control of severely rotated premolars [78]. Other ex vivo and clinical trials have argued for larger and sharper-edged attachments to improve aligner engagement [64,79,80]. Clinically, some authors reported that anterior rotations can be effectively managed without attachments, and that placing them only on the rotated tooth, rather than on adjacent teeth, may increase efficiency [62,81]. Overall, attachments remain useful for specific scenarios—such as short crowns or rotations greater than 15°—but their true impact on predictability remains uncertain.

#### 4.1.3. Interproximal Reduction (IPR)

Interproximal reduction is a common adjunct in clear aligner therapy, intended to create space, reduce interproximal resistance, and facilitate tooth alignment [12,21]. It is typically recommended in cases of moderate crowding or when substantial rotations are required.

Most of the included studies reported using IPR [38,39,40,41,42,43,46,47], though with substantial variability. Some authors applied standardized protocols, often limiting enamel reduction to <1 mm per arch [38] or restricting it to anterior teeth [41]. Others implemented individualized protocols tailored to the clinical condition of each patient, without specifying the extent and location of IPR [40,46,47]. Maree et al. [44] explicitly excluded its use.

Despite its widespread application, the specific contribution of IPR to improved rotational accuracy remains inconclusive. Among the analyzed articles, only Kravitz et al., isolated IPR as a variable, comparing groups with and without enamel reduction. Although the group receiving IPR mesial and distal to canines achieved the highest mean rotational accuracy (43%) and the lowest standard deviation (SD = 22.6%), differences were not statistically significant [42]. Several authors still reported acceptable rotational outcomes with individualized IPR protocols [39,40,43], while others, such as Maree et al. [44], obtained satisfactory results in the absence of IPR, particularly in maxillary incisors. Similarly, Ghislanzoni [41] found no significant influence of IPR on incisor and canine rotations.

This variability among findings reflects a broader inconsistency in the literature. While some authors advocate IPR as a key factor in enhancing rotational efficiency [57,67], others report no significant benefit [52,62]. Current evidence does not consistently confirm IPR effectiveness in improving rotational accuracy.

#### 4.1.4. Staging

In clear aligner therapy, staging refers to the breakdown of planned tooth movements into sequential steps, each corresponding to a single aligner. It defines both the magnitude of movement per tray and the temporal progression of treatment [5,82]. From a biomechanical standpoint, accurate staging during the digital planning phase is essential to optimize force systems, minimize aligner deformation, and reduce tracking errors, especially for complex movements like rotations [12,21,74,83].

In this review, eight of the twelve included studies explicitly reported staging protocols. Most adopted a standardized rotational rate of approximately 2° per aligner [39,43,44,46,47], consistent with Invisalign^®^ default settings [84]. Ghislanzoni et al. [41] and Medeiros et al. [45] described longer protocols, with an average use of 14 and 24 aligners, respectively, while Al-Nadawi et al. [38] and D’Antò et al. [40] did not specify rotation per stage.

Literature findings emphasize that reducing staging to less than 1.5° per aligner improves rotational accuracy [21,85]. Simon et al., demonstrated that premolar rotations staged at <1.5°/aligner achieved a mean efficacy of 41.8%, compared to only 23% when staged at >1.5°/aligner [74]. Rossini et al., similarly recommended limiting increments to under 1.5°, especially for round-shaped teeth [2]. More recently, Ferlias et al., and Cortona proposed even lower thresholds (1° or 1.2°/aligner), suggesting these values better align with biological force systems and ensuring consistent moment delivery [64,86]. These findings have been corroborated by finite element analyses [81].

Beyond magnitude, the sequence and timing of tooth movements—commonly referred to as macro- and micro-staging—also play a critical role in treatment success [69,83]. Macro-staging involves anchorage planning, while micro-staging coordinates the timing of individual tooth movements. Strategies such as round-tripping (e.g., initial expansion and proclination followed by rotation and retraction) or hinge rotations—instead of pure rotation—can enhance rotational predictability by creating space and reducing friction [21,51]. Among the reviewed studies, only Maree et al. [44] discussed a strategic staging sequence, recommending early mesiodistal uprighting of incisors, thereby increasing interproximal space and facilitating subsequent rotation. This approach highlights the value of early space creation to enhance the efficiency of later rotational movements.

Furthermore, the direction of rotation appears to influence treatment outcomes. Mesial rotations have shown greater predictability compared to distal ones, likely due to lower moments required and more favorable force vectors [22,67,87].

#### 4.1.5. Materials and Aligner System Used

The material composition of clear aligners is a key determinant of their biomechanical performance. The mechanical properties of the polymers—including elasticity, load-deflection behavior, shape memory, and stress relaxation—determine how effectively forces are transmitted to the teeth [3,21,87]. SmartTrack^®^ (Align Technology), a multilayer polyurethane with improved elastic recovery, has demonstrated superior force maintenance and reduced stress decay compared to earlier materials such as EX30 [5,21]. While the latter, a stiffer single-layer polymer, was associated with higher initial rigidity and may favor specific movements like buccolingual tipping, SmartTrack^®^ offers more consistent force delivery over time [88]. By contrast, thermoformed aligners made of PETG or polyurethane tend to deform and dissipate force, limiting their effectiveness for rotational control [86,89].

Across the included studies, most employed Invisalign^®^ aligners, either EX30 [42], SmartTrack^®^ [38,44], or both [45]. The F22 system, composed of a polyurethane with favorable stress relaxation properties [90], was evaluated in two articles [43,46]. Other systems included Ordoline [40], 3M Clarity [49], and Flash aligners [48], while Sachdev et al. were the only study group to employ in-office 3D-printed aligners [47]. However, few studies directly assessed the relationship between material characteristics and rotational accuracy, limiting definitive conclusions.

Growing interest in alternative fabrication technologies, such as direct 3D printing and shape-memory polymers, further highlights the need for advances in aligner material science. For instance, direct-printed aligners using TC-85 (Graphy^®^, Seoul, Republic of Korea) have shown superior dimensional stability and force consistency compared to conventional PETG, though clinical evidence remains preliminary [89,91]. Similarly, recent finite element studies suggest that aligner thickness and elastic modulus significantly influence rotational predictability [81]. Current evidence supports the notion that advancements in aligner material composition—particularly those aimed at reducing deformation and improving elastic recovery—could play a significant role in enhancing the biomechanical efficiency of rotational movements.

#### 4.1.6. Aligner Wear Protocols

Aligner wear protocols influence both the biomechanical effectiveness of orthodontic forces and the overall efficiency of CAT. Most included studies adopted either 7-day or 14-day regimens, with some allowing variation based on clinical judgment [38,39,41,42]. Al-Nadawi et al., reported no significant differences among 7-, 10-, and 14-day protocols for anterior tooth movements, suggesting that shorter schedules may reduce overall treatment time without compromising outcomes [38]. On the other hand, Castroflorio et al. indicated that posterior rotations may benefit from 14-day intervals to improve control [39], while Kravitz et al., suggested that complex movements may require even longer wear, in some cases up to 21 days [42].

From a biological standpoint, the majority of orthodontic tooth movement occurs within the first week of aligner wear [8,92,93]. Nevertheless, force decay due to stress relaxation in thermoplastic materials supports shorter protocols [87]. Concerns remain, however, regarding inadequate periodontal recovery with accelerated schedules [9,94]. Recent studies advocate for a personalized or “hybrid” approach, with 7-day intervals for simple movements and longer durations for complex ones such as rotations or torque in posterior segments [95]. Randomized trials comparing 10- and 14-day protocols found no significant differences in movement efficacy or patient discomfort, further supporting flexibility in regimen selection based on case complexity [96].

#### 4.1.7. Additional Strategies to Enhance Rotational Predictability with CAT

Despite advancements in clear aligner therapy, rotational movements remain among the most challenging to predict and control. A frequently discussed limitation concerns the reliability of digital treatment planning software. As noted by Al-Nadawi et al., ClinCheck^®^ represents a force-based simulation rather than a precise predictor of final tooth position, emphasizing the need for clinician vigilance and ongoing refinement during treatment [38]. Similarly, Castroflorio et al., highlighted that current software systems cannot account for individual biological variability, making final virtual setups only an approximation of clinical outcomes [39].

Another key consideration is the magnitude of prescribed movement. Larger derotations, particularly >15° and in rounded teeth, are generally less accurate [46,71,74]. To address this, many authors recommend planning overcorrections during digital setup, with suggesting values ranging from 5° to 10° [2,52,82,97]. Palone et al. observed that an average of 28.4% of the initially prescribed rotational movement required correction during the finishing phase, suggesting that overcorrection may need to exceed 20% for movements with inherently poor accuracy, such as premolar derotations [98]. Similarly, Wen et al., proposed overcorrections of 27% for maxillary incisors, 30% for mandibular incisors, 35% for maxillary canines, and 38% for maxillary premolars [99]. Additionally, Kravitz et al., proposed implementing overcorrections during the finishing phase rather than throughout the entire treatment [42].

It is important to note that refinements and auxiliaries were not considered in this review to avoid introducing confounding factors; however, their clinical impact on rotational outcomes is well documented in the literature. When derotating teeth, up to 70–80% of patients require refinement aligners to achieve acceptable accuracy [53,57,100].

Finally, auxiliary tools—including elastics, bonded buttons, sectional wires, and TADs—can further enhance control [59,97,101,102,103]. The “hybrid approach”, combining clear aligners with fixed appliances or cantilever systems, has emerged as a promising strategy to increase predictability while minimizing reliance on extensive aligner sequences [60,104]. Incorporating such adjunctive measures into clinical protocols may help overcome the inherent biomechanical limitations of aligner-only therapies, particularly in challenging rotational corrections.

### 4.2. Study Limitations

This review presents some limitations. Although the included studies provided meaningful insights into the rotational performance of clear aligners, their methodological quality and certainty of evidence—assessed through RoB 2, ROBINS-I, and GRADE [33,34,35,36,37]—were limited. Nearly all investigations were judged to be at moderate to serious risk of bias in at least one domain, mainly due to confounding, patient selection, and difficulties in objectively verifying compliance. Moreover, the certainty of evidence was rated as low for all non-randomized studies and only moderate for the single available RCT. These factors temper the strength of the conclusions, indicating that the reported outcomes should be regarded as indicative rather than definitive.

The evidence base was further constrained by the predominance of non-randomized designs, with only one RCT available. This reflects a broader trend in aligner research, where systematic reviews are increasing while high-quality primary trials remain scarce [27,105]. Retrospective or in vitro studies are predominant; while they may provide valuable insights, these designs are inherently more prone to bias and often lack the contextual relevance required for real-world application [27].

Another limitation is the marked methodological heterogeneity across the included investigations, particularly regarding study design, sample size, tooth types assessed, and measurement software. This variation precluded meta-analytic synthesis and further reduced the overall strength of the evidence.

In addition, our review focused exclusively on studies employing 3D digital model superimpositions to compare predicted and achieved tooth positions. While this method is widely used and generally considered accurate [40,47,106], its reliability can vary depending on the reference areas selected and the extent of tooth displacement during treatment [27,43].

Furthermore, no standardized protocols were applied to assess and control patient compliance, a factor that substantially influences treatment outcomes [56,71]. Finally, only adult patients were considered, excluding adolescents due to their differing bone remodeling dynamics and potentially reduced compliance [46,107,108]. While this ensured greater homogeneity, it limits the generalizability of our findings.

### 4.3. Future Directions

To address current gaps in knowledge, future research should prioritize high-quality, prospective randomized controlled trials with standardized outcome measures to clarify factors influencing rotational accuracy in CAT [91]. The adoption of consistent definitions of accuracy and tooth-specific analyses would enable more reliable quantitative comparisons across studies. Comparative investigations between aligner systems and materials—including emerging in-office 3D printed options—are also needed, alongside trials to establish thresholds for when attachments and IPR become essential to achieve effective rotational control.

Validated and standardized model superimposition methods, ideally combined with skeletal landmarks, should be employed to minimize methodological bias [52]. Moreover, multivariate analyses considering patient-related variables such as age, sex, bone density, and compliance are necessary to support more individualized treatment planning. Finally, future studies should include more diverse populations, particularly adolescents, and report outcomes separately for cases involving refinements or auxiliaries. Such efforts would provide a more realistic evaluation of rotational performance in clinical practice and contribute to the development of optimized, evidence-based treatment protocols.

## 5. Conclusions

This systematic review provides an updated analysis of the evidence on the rotational efficacy of clear aligner therapy (CAT) in adult patients, focusing exclusively on studies that employed 3D superimposition for outcome assessment.

Key findings are as follows:

- Overall, rotational accuracy was suboptimal across studies, with considerable variability across studies and no investigation reporting complete correspondence between planned and achieved rotation. However, a progressive improvement in performance was observed in more recent studies, likely related to advances in materials, digital planning, and clinical protocols.
- Tooth-specific performance varies significantly, with incisors and molars showing generally higher accuracy compared to “round-shaped teeth”, especially maxillary canines and premolars.
- The role of attachments and interproximal enamel reduction (IPR) is potentially relevant, but current evidence remains inconclusive. Considerable heterogeneity in study protocols and insufficient standardization prevent reliable comparisons and preclude definitive conclusions on the isolated contribution of each factor.
- Staging emerges as a critical factor in improving rotational control: values below 1.5° per aligner are associated with enhanced accuracy, although most studies employed a staging of approximately 2° per stage.
- Other strategies, such as planned overcorrections and refinements, may enhance rotational control but require further validation.

Given that nearly all included studies presented moderate-to-serious risk of bias and low certainty of evidence, these results should be interpreted cautiously. At present, the findings are best regarded as indicative trends rather than definitive guidance.

In conclusion, while the predictability of rotational movements with CAT shows gradual improvement, further well-designed, controlled studies with standardized outcome measures are still needed to establish reliable clinical guidelines and strengthen evidence-based decision-making.

## Figures and Tables

**Table 1:** Search strategy for the seven databases (PubMed, Scopus, Web of Science, Embase, Cochrane, LILACs and Ovid) and the two grey literature portals.

Database	Search Strategy	Results
**PubMed**	(((((aligner*) OR (invisalign)) OR (spark)) OR (clearcorrect)) AND ((tooth movement[MeSH Terms]) OR (rotation[MeSH Terms]) OR (rotation*) OR (“tooth movement*”))) AND (((((((((treatment outcome[MeSH Terms]) OR (accuracy)) OR (predictability)) OR (predict*)) OR (efficacy)) OR (“planned movement*”)) OR (outcome*)) OR (discrepancy)) OR (efficacy)) Sort by: Most Recent	674
**Scopus**	(TITLE-ABS-KEY ((aligner* OR invisalign OR spark OR clearcorrect))) AND (TITLE-ABS-KEY (“tooth movement*” OR rotation* OR “orthodontic movement*”)) AND (TITLE-ABS-KEY (accuracy OR predictability OR predict* OR efficacy OR “planned movement*” OR outcome* OR discrepancy))	582
**Embase**	(‘clear aligner*’ OR aligner* OR ‘invisalign’ OR ‘invisalign’/exp OR invisalign OR ‘spark’ OR ‘spark’/exp OR spark OR clearcorrect) AND (rotation* OR ‘tooth movement’/exp OR ‘tooth rotation’ OR ‘tooth rotation*’ OR ‘tooth movement*’ OR ‘orthodontic movement*’) AND (‘treatment outcome’/exp OR accuracy OR predictability OR predict* OR efficacy OR ‘planned movement*’ OR outcome* OR discrepancy)	415
**Web Of Science**	#1 ALL = (aligner* OR “clear aligner*” OR invisalign OR spark OR “clearcorrect”) #2 ALL = (rotation* OR “tooth rotation*” OR “tooth movement*” OR “orthodontic movement*”) #3 ALL = (“treatment outcome*” OR accuracy OR predictability OR predict* OR efficacy OR “planned movement*” OR outcome* OR discrepancy) #1 AND #2 AND #3	594
**Cochrane Library**	#1 aligner* OR (clear NEXT aligner*) OR invisalign OR spark OR “clearcorrect” #2 rotation* OR (tooth NEXT rotation*) OR (tooth NEXT movement*) OR (orthodontic NEXT movement*) #3 (treatment NEXT outcome*) OR accuracy OR predictability OR predict* OR efficacy OR (planned NEXT movement*) OR outcome* OR discrepancy #1 AND #2 AND #3	65
**LILACs**	(aligner* OR “clear aligner*” OR invisalign OR spark OR “clearcorrect”) AND (rotation* OR “tooth movement*” OR “tooth rotation*” OR “orthodontic movement*”) AND (“treatment outcome*” OR accuracy OR predictability OR predict* OR efficacy OR “planned movement*” OR outcome* OR discrepancy)	33
**Ovid**	#1 (aligner* or “clear aligner*” or Invisalign or Spark or “ClearCorrect”).mp. #2 Tooth Movement/or Rotation/or “tooth movement*”.tw. or rotation*.tw. or “orthodontic movement*”.tw. #3 Treatment Outcome/or “treatment outcome*”.tw. or accuracy.tw. or efficacy.tw. or predict*.tw. or “planned movement*”.tw. or outcome*.tw. or discrepancy.tw. #4 #1 AND #2 AND #3	240
**Clinicaltrials.gov**	(clear aligner OR Invisalign OR aligner OR “clear aligners” OR Spark OR ClearCorrect) AND (tooth movement OR rotation OR orthodontic movement OR dental rotation) AND (accuracy OR efficacy OR predictability OR treatment outcome)	41
**Proquest.com**	abstract(clear aligner OR Invisalign OR aligner OR “clear aligners” OR Spark OR ClearCorrect) AND abstract(tooth movement OR rotation OR orthodontic movement OR dental rotation) AND abstract(accuracy OR efficacy OR predictability OR treatment outcome)	94

**Table 3:** Data about the primary outcome of the present review. Description of the aligner system and the superimposition software used to compare initial, planned and final outcome. Mean predicted and achieved rotational movement. Indicators of clear aligner efficiency: percentage accuracy, Lack of Correction (LC), Mean Absolute Error (MAE).

First Author, Year (Reference)	Aligner System Used	Teeth Assessed	Superimposition Software	Mean Planned Rotation (°)	Mean Achieved Rotation (°)	Percent Accuracy	LC	MAE
Al-Nadawi 2021 [38]	Invisalign (SmartTrack)	From central incisors to second molars, Mx and Mb	eModel Compare 9.0	N/R	N/R	N/R	Group A: 2.88° Group B: 2.86° Group C: 2.54°	N/R
Castroflorio 2023 [39]	Invisalign	From central incisors to second molars, Mx and Mb	Geomagic Qualify	7.24°	N/R	N/R	2.93°	N/R
D’Antò 2024 [40]	Ordoline aligners	From central incisors to second molars, Mx and Mb	Geomagic Control X	8.9°	6.5°	76.8%	N/R	2.34°
Ghislanzoni 2024 [41]	Invisalign	From central incisors to first molars, Mx and Mb	VAM Software	2.54°	1.56°	N/R	N/R	2.3°
Kravitz 2008 [42]	Invisalign	Canines, Mx and Mb	ToothMeasure	11.8°	N/R	Group AO: 33.3 ± 28.6% Group IO: 43.1 ± 22.6% Group N: 30.8 ± 27.3%	N/R	N/R
Lombardo 2017 [43]	F22 aligners	From central incisors to second molars, Mx and Mb	VAM Software	8°	3.77°	68.1%	4.42°	N/R
Maree 2022 [44]	Invisalign (SmartTrack)	Central incisors, Mx	Geomagic Control X	18.75°	13.37°	71.3%	N/R	N/R
Medeiros 2024 [45]	Group A: Invisalign EX30 Group B: Invisalign (SmartTrack)	From incisors to molars	Geomagic Control	Total: 7.8° Group A: 8.09° Group B: 7.7°	Total: 3.3° Group A: 3.75° Group B: 3.22°	N/R	Total: 4.45° Group A: 4.34° Group B: 4.48°	N/R
Mario 2024 [46]	F22 aligners	From central incisors to first molars, Mx and Mb	Onyxceph 3TM	8.3°	4.81°	61.6%	4.18°	N/R
Sachdev 2021 [47]	In-office 3D direct-printed aligners	From central incisors to canines, Mx and Mb	OrthoAnalyzer TM	6.34°	3.13°	50.1%	3.21°	N/R
Sorour 2022 [48]	Group A: Invisalign Group B: Flash	From central incisors to canines, Mx and Mb	eModel Compare 8.1	N/R	N/R	N/R	Group A: 3.2° Group B: 3.1° Total: 3.2°	N/R
Taebi-Harandy 2023 [49]	3M Clarity	From central incisors to second bicuspid, Mx and Mb	3D Slicer Version 4.11	4.84°	2.72°	N/R	0.74°	N/R

N/R: not reported.

**Table 4:** Information related to the secondary outcomes of the review.

First Author, Year (Reference)	Attachments	IPR	Rotation Rate (°/Aligner)	Mean n° of Aligners	Days of Aligner Wear	Average Treatment Duration
Al-Nadawi 2021 [38]	Individualized per patient Average of 6 attachments/arch	Yes, <1 mm/arch on average	N/R	Group A: 20 Group B: 21 Group C: 20	Group A: 7 days Group B: 10 days Group C: 14 days	Group A: 5 months Group B: 8 months Group C: 9 months
Castroflorio 2023 [39]	3 configurations: No attachments Conventional attachments Optimized attachments	Individualized per patient	2°/aligner	Mx: 27 ± 15 Mb: 25 ± 11	7 to 14 days	9.8 ± 3.8 months
D’Antò 2024 [40]	Conventional attachments Laterals: sash attachments Canines, 1st and 2nd premolars: rectangular vertical 1st and 2nd molars: rectangular horizontal	Yes, allowed	N/R	N/R	10 days	N/R
Ghislanzoni 2024 [41]	Optimized attachments automatically placed by the ClinCheck software No attachments on molars	Yes, if needed, only on anterior teeth	N/R	14	14 days	7 months
Kravitz 2008 [42]	Group AO: attachments (70.5% vertical ellipsoid, 0.75 mm thick, labially placed and centrally located) Groups IO and N: no attachments	Groups AO and N: no Group IO: yes	N/R	10 Mx, 11 Mb	14 to 21 days	7.2 months
Lombardo 2017 [43]	F22 system Grip Points	Yes, allowed	2°/aligner	N/R	14 days	N/R
Maree 2022 [44]	Different geometries: Optimized extrusions Optimized root uprighting Rectangular vertical Horizontal ovoid	No	2°/aligner	N/R	14 days	N/R
Medeiros 2024 [45]	Yes, but not specified	N/R	N/R	24	14 days	11.2 months
Palone 2024 [46]	F22 system Grip Points, triangular shaped. Used for derotation ≥ 10° of rounded teeth and >20° for Mb incisors and Mx lateral incisors	Yes, allowed	2°/aligner	15	14 days	N/R
Sachdev 2021 [47]	Only for derotations ≥5°, according to the attachments protocol	Yes, allowed	2°/aligner	7	14 days	N/R
Sorour 2022 [48]	Yes Invisalign: average of 6 attachments/arch Flash: average of 11 attachments/arch	N/R	N/R	Group A: 21 Mx, 20 Mb Group B: 21 Mx, 21 Mb	10 days	Group A: 8.4 months Group B: 6.9 months
Taebi-Harandy 2023 [49]	N/R	N/R	N/R	N/R	N/R	N/R

Mb: mandibular; Mx: maxillary; N/R: not reported.

## Data Availability

The data presented in this study are available in the article.

## Supplementary Materials

The following supporting information can be downloaded at https://www.mdpi.com/article/10.3390/dj13100440/s1, Table S1: Grading of Recommendation, Assessment, Development, and Evaluation (GRADE) analysis; Table S2: Summary of Findings.
