# Supplementary material for: Evaluating the Clinical Success of Clear Aligners for Rotational Tooth Movements in Adult Patients: A Systematic Review
Source: Dent J (Basel). 2025 Sep 24;13(10):440. doi: 10.3390/dj13100440 (PMC12564582; doi:10.3390/dj13100440)
Supplement: Supplementary file 1 [file dentistry-13-00440-s001.zip › dentistry-3841059-supplementary.pdf]

Evaluating the Clinical Success of Clear Aligners for Rotational Tooth Movements in Adult Patients: A Systematic Review

Giulia Benedetti [1], Nicolò Sicca [1], Gaia Lopponi [1], Claudia Dettori [2], Alessio Verdecchia [1,3,*] and Enrico Spinas [1,*]

1 Department of Surgical Sciences, Postgraduate School in Orthodontics, University of Cagliari, 09124 Cagliari, Italy; giulia.benedetti995@gmail.com (G.B.); n.sicca@studenti.unica.it (N.S.); gaia.lopponi@gmail.com (G.L.)
2 Department of Surgical Sciences, School of Dental Medicine, University of Cagliari, 09124 Cagliari, Italy;
claudia.dettori@gmail.com
3 Orthodontics Division, Instituto Asturiano de Odontologia, Universidad de Oviedo, 33006 Oviedo, Spain
* Correspondence: verdecchia.belli.a@gmail.com (A.V.); enricospinas@tiscali.it (E.S.);
Tel.: +33-778-90-40-53 (A.V.); +39-330-412295 (E.S.)

Supplementary information

Table S1 - Grading of Recommendation, Assessment, Development, and Evaluation (GRADE) analysis.

**Certainty of assessment**							**Certainty**
Nº of studies	Study design	Risk of bias	Inconsistency	Indirectness	Imprecision	Other considerations	
**Mean planned rotation and mean achieved rotation**							
**12** [30; 31; 32; 33; 34; 35; 36; 37; 38; 39; 40; 41]	1RCT<br>5 P<br>6 R	Serious	Serious	Not serious	Not serious	None	Low<br>⨁◯◯◯
**Use of attachments**							
**11** [30; 31; 32; 33; 34; 35; 36; 37; 38; 39; 40]	1RCT<br>5 P<br>5 R	Serious	Serious	Not serious	Not serious	None	Low<br>⨁◯◯◯
**Use of IPR**							
**7** [30; 31; 32; 33; 34; 35; 38; 39]	1RCT<br>5 R<br>2 P	Serious	Serious	Not serious	Not serious	None	Low<br>⨁◯◯◯

RCT = Randomized Clinical Trials; P = Prospective study; R = Retrospective study

GRADE Working Group grades of evidence. High quality: Further research is very unlikely to change our confidence in the estimate of effect. Moderate quality: Further research is likely to have an important impact on our confidence in the estimate of effect and may change the estimate. Low quality: Further research is very likely to have an important impact on our confidence in the estimate of effect and is likely to change the estimate. Very low quality: We are very uncertain about the estimate.

Table S2 – Summary of Findings (SoF) Table

Outcome	No. of studies	Findings (range)	Certainty of evidence (GRADE)	Comments
**Percent accuracy**	6	36% – 77%	Low	High heterogeneity: accuracy depends on tooth type
**Lack of Correction (LC)**	8	0.7° – 4.5°	Low	Lower values indicate higher accuracy
**Mean Absolute Error (MAE)**	2	2.3°	Low	
**Tooth type differences**	11	Incisors & molars: higher accuracy; Canines & premolars: least predictable	-	Consistent trend across studies
**Attachments**	11	Widely used, but scarce design description and inconsistent results on their efficacy	Low	Designs varied; contribution unclear
**Interproximal reduction (IPR)**	9	Commonly applied, but often not quantitatively described; impact inconsistent	Low	No standardized protocols described
**Staging protocols**	5	2°/aligner	-	-
**Aligner wear schedules**	11	Most adopted 7–14 days; some up to 21 days	-	-
